# The Development of a New Sport-Specific Classification of Coping and a Meta-Analysis of the Relationship between Different Coping Strategies and Moderators on Sporting Outcomes

**DOI:** 10.3389/fpsyg.2016.01674

**Published:** 2016-11-03

**Authors:** Adam R. Nicholls, Natalie J. Taylor, Sean Carroll, John L. Perry

**Affiliations:** ^1^Department of Sport, Health, and Exercise Science, University of HullHull, UK; ^2^Centre for Healthcare Resilience and Implementation Science, Australian Institute of Health and Innovation, Macquarie UniversitySydney, NSW, Australia

**Keywords:** age, gender, goal attainment, objective performance, subjective performance

## Abstract

There is an ever growing coping and sports performance literature, with researchers using many different methods to assess performance and different classifications of coping. As such, it makes it difficult to compare studies and therefore identify how coping is related to performance. Furthermore, there are no quantitative syntheses of the results from these studies. A quantitative synthesis would facilitate a more comprehensive understanding of how coping is associated with athletic performance. In order to accurately compare studies, our first aim was to develop a new coping classification that would make this possible. Firstly, we reviewed the strengths and limitations of the different coping classifications and then identified the commonalities and differences between such classifications. We opted for a three-factor classification of coping, because the evidence suggests that a three-factor classification provides a superior model fit to two-factor approaches. Our new classification of coping was based on an existing model from the developmental literature, which received an excellent model fit. We made some adaptations, however, as our classification was intended for an athletic population. As such, we classified coping as mastery (i.e., controlling the situation and eliminating the stressor), internal regulation (i.e., managing internal stress responses), or goal withdrawal (i.e., ceasing efforts toward goal attainment). Undertaking a meta-analysis, our second aim was to identify which coping strategies correlated with sports performance and whether this relationship varied according to moderator variables. Articles were sourced from online electronic databases and manual journal searches. PRISMA guidelines were used to search, select, and synthesize relevant studies. Random effects meta-analyses were performed to identify associations between coping classification and sport performance. *Q, I*^2^, and *R*^2^ values assessed heterogeneity. Eighteen published investigations, including 3900 participants and incorporating fifty-nine correlations, indicated an overall positive effect for mastery coping, a negligible negative effect for internal regulation coping, and a negative effect for goal withdrawal strategies. The findings of this meta-analysis could be used by sports practitioners to help them deliver effective coping interventions. In order to maximize performance, practitioners could encourage the use of mastery coping, but advise their athletes not to use goal withdrawal strategies.

## Introduction

Playing sport can be a very stressful experience, with athletes reporting a wide range of performance-related, coach-related, and expectations from others (Nicholls and Levy, 2016b). Coping is a self-regulatory mechanism that enables athletes to manage stressors, and includes all voluntary thoughts and behaviors aimed at managing internal or external demands that have been appraised as being stressful (Lazarus and Folkman, 1984). It includes individual or combined attempts to cope (i.e., athlete coping by him or herself, or a coach and athlete coping together; Nicholls and Perry, 2016a). Researchers such as Oliver et al. (2010) suggested that coping may be related to whether youth athletes are successful in reaching their full potential, making the transition from academy level to first team (Finn and McKenna, 2010), resilience (Nicholls et al., 2016a), and even the coach-athlete relationship (Nicholls et al., 2016b). All of these factors may contribute to athletic performance. Indeed, there is a growing interest in the relationship between coping and sports performance. Comparing studies, to help practitioners identify the most effective coping strategies to teach their athletes, is problematic due to the different ways in which both coping and sports performance are often measured or classified. Therefore, we wanted to clarify this relationship by devising a new classification of coping and then using a meta-analysis to quantify the relationship.

### Coping classifications

Three methods of classifying coping are widely used within the sport psychology literature (e.g., Lazarus and Folkman, 1984; Roth and Cohen, 1986; Gaudreau and Blondin, 2004a). Lazarus and Folkman (1984) classified coping within problem- and emotion-focused dimensions. Problem-focused coping refers to strategies that manage or eliminate the stressor causing the distress, whereas emotion-focused coping refers to coping strategies that regulate emotional responses to the stressor. Roth and Cohen (1986) categorized coping into approach or avoidance strategies. Approach coping involves the athlete confronting the stressor then eliminating it by taking direct action (e.g., developing a plan of action or instilling more effort). Conversely, avoidance coping involves psychological and/or behavioral attempts at disengaging from a stressful situation.

It is important to note that scholars criticized Lazarus and Folkman's (1984) and Roth and Cohen's (1986) classifications of coping. Ayers et al. (1996), Connor-Smith et al. (2000), and Walker et al. (1997) all found evidence to suggest that these classifications of coping do not adequately reflect the structure of coping. In particular, Ayers et al. reported that active coping included both problem- and emotion-focused coping, whereas Connor-Smith et al. found that problem solving loaded with two sub-types of emotion-focused coping strategies. These findings support Coyne and Gottlieb's (1996) earlier contention that the problem- and emotion-focused coping dimensions are too broad and that different types of coping are put into the same category. Further, Compas et al. (1996) stated that some individual coping strategies may serve both a problem- and emotion-focused coping function simultaneously. Similar to the problem- and emotion-focused dimensions of coping, the approach, and avoidance classification of coping also received additional criticism for being too broad and not being able to distinguish between subtypes of coping (Compas et al., 2001).

In order to address some of the aforementioned limitations, and based upon the three-factor approach adopted by Connor-Smith et al. (2000) and Walker et al. (1997), Gaudreau and Blondin (2004a) developed a sport-specific approach to classification of coping. These authors classified coping as task-oriented (i.e., attempts to master a stressful situation), disengagement-oriented (i.e., no longer attempting to strive for personal goals), or distraction-oriented coping (i.e., focusing on cues that are not sport relevant). Although, the approach by Gaudreau and Blondin is much more likely to capture the structure of coping than other classifications used in the sport literature (e.g., Ayers et al., 1996; Walker et al., 1997; Connor-Smith et al., 2000; Compas et al., 2001), the widespread use of classifying coping in different way makes it difficult to compare the coping and performance relationship across different studies. This is because there is some overlap between the three classifications so widely used in the sport literature. For instance, although task-oriented coping is similar to problem-focused coping, task-oriented coping also features an element of emotion-focused coping. Gaudreau and Blondin's task-oriented dimension includes seeking support and relaxation, which would be classified as emotion-focused coping within Lazarus and Folkman's (1984) framework. This overlap between the different classifications of coping causes ambiguity when attempting to compare studies that utilize different ways of categorizing coping (Compas et al., 2001), and highlights why a new classification is required if one wants to compare studies that utilized different ways of categorizing coping.

### Sport performance

There is empirical evidence regarding the relationship between coping and: (a) objective performance, (b) subjective performance, and (c) a combination of both subjective and objective sports performance measures. Objective measures of performance used in coping research include actual performance scores, such as the number of shots taken in golf (Gaudreau et al., 2010), batting average in baseball (Smith and Christensen, 1995), or performance time on a rowing test (Hatzigeorgiadis, 2006). Subjective assessments of performance involve athletes reporting the extent to which they are satisfied with their performance on a Likert-type scale (Nicholls et al., 2012a), goal achievement (Schellenberg et al., 2013), or a combination of both objective and subjective assessments to indicate a measure of performance (Gaudreau and Blondin, 2004b).

### Coping and objectively measured sports performance

Smith and Christensen (1995) examined the relationship between athlete- and coach-assessed coping along with batting and pitching seasonal averages among professional baseball players, during the 1991 season. Interestingly, the players' ratings of coping correlated less with performance than coach ratings of coping. The strategy confidence and achievement motivation correlated positively with performance for both batters and pitchers. Alternatively, peaking under pressure correlated with pitchers' performance, but not the batters. Haney and Long (1995) examined the relationship between coping and the number of baskets or field goals scored across two rounds of performance. Engagement coping positively predicted performance in both Round 1 and Round 2, whereas disengagement coping negatively predicted performance in Round 1 and Round 2.

Hatzigeorgiadis (2006) examined the relationship between coping and performance across two indoor rowing tests. After the initial 500 m test, participants were randomly split into two groups and provided with target times for a 3000 m rowing test. In one group the rowers had little chance of achieving their set goal (low-goal attainment expectancy; Low GAE), whereas the rowers in the other group had a high chance of achieving their goal (high-goal attainment expectancy; High GAE). There was no change in tempo for those in the Low GAE group throughout the whole trial, whereas there was an increase in tempo for those in the High GAE group from 2500 to 3000 m of the trial. Rowers in the High GAE group reported significantly more effort, but significantly less behavioral disengagement and mental disengagement than those in the Low GAE group. With a sample of 54 golfers, who completed measures of coping after six rounds, Gaudreau et al. (2010) found an association between task-oriented coping and the golfers' superior performance, whereas disengagement-oriented coping was related to inferior performance. Doron and Gaudreau (2014) linked winning streaks in fencing to fencers using more task-oriented coping, in comparison with losing streaks and non-streaks.

In contrast to the aforementioned research, Van Yperen (2009) used a different approach to assess objective performance within professional soccer. Players reported their coping when they were members of a soccer academy. Players were classified as being successful if they forged a career in professional soccer player or unsuccessful if they did not become a professional soccer player within 15 years of the initial coping data collection. Those who progressed into professional football scored significantly higher on problem-focused coping and used more social support coping strategies than those who did not progress to a professional club.

### Coping and subjectively measured performance

Scholars (Levy et al., 2011; Nicholls et al., 2012a; Laborde et al., 2014a,b) assessed the relationship between coping reported at the dimensional level and subjective performance, whereas Nicholls et al. (2012b) examined the relationship between coping strategies and subjective performance. Task-oriented coping correlated positively with subjective performance, whereas disengagement-oriented coping was negatively associated with performance (Levy et al., 2011; Nicholls et al., 2012a; Laborde et al., 2014b). Laborde et al. (2014a) also found a significant and positive correlation between subjective performance and task-oriented coping, although the relationship between subjective performance and both distraction- and disengagement-oriented coping were both insignificant. Nicholls et al. (2012a) reported a significant and negative relationship between distraction-oriented coping and performance satisfaction, whereas Levy et al. did not. Nicholls et al. (2012b) reported positive and significant relationships between performance satisfaction and mental imagery, effort expenditure, thought control, relaxation, and logical analysis, but a negative relationship with disengagement/resignation.

Gaudreau and Blondin (2004a) purported that goal attainment can also be used as a subjective measure of performance, because it generally includes the criteria upon which athletes assess their individual performance. Measures used to assess goal attainment include the 12-item Attainment of Sport Achievement Goal Scale (Gaudreau and Amiot, manuscript submitted), a 3-item scale (Gaudreau et al., 2010), a 2-item scale (Gaudreau and Blondin), or a sliding scale anchored at −5 (negative feeling about ability to achieve goal) and +5 (positive feelings about ability to achieve goal; Evans et al., 2014). The majority of these studies (e.g., Amiot et al., 2004; Gaudreau and Antl, 2008; Nicolas et al., 2011; Schellenberg et al., 2013) found a positive association between goal attainment and task oriented coping, but a negative relationship between goal attainment and disengagement-oriented coping. Distraction-oriented coping was not associated with goal attainment in these studies. Evans et al. (2014) explored how athletes coped when they were in danger of not achieving their goals and therefore experienced concerns about performance. The endurance athletes in this study used problem-focused and emotion-focused coping strategies when they had negative feelings about their goals.

### Combined performance assessment

Gaudreau and colleagues (e.g., Gaudreau et al., 2002; Gaudreau and Blondin, 2004b) devised a method to assess performance that included both subjective and objective indicators, which they called performance-goal discrepancy. Golfers were instructed to set a realistic score for an upcoming round and then their actual score was subtracted after the round had finished. The subjective element involved the creation of the goal and an objective element included the golfers' score. Seeking social support, active coping/planning, suppression of competing activities, and positive reappraisal during competitive play were associated with golfers performing better. Conversely, behavioral disengagement and humor were associated with golfers performing more poorly (Gaudreau et al., 2002). Gaudreau and Blondin reported an association between task-oriented coping and golfers performing better in relation to their goal. Disengagement-oriented coping was associated with players not performing well in relation to their goals.

### Moderators of the coping and performance relationship

It is important to explore study characteristics that may affect the strength and or the direction of the relationship between coping and performance, because this may help explain any inconsistent findings. The literature indicates that there are gender (e.g., Nicholls et al., 2009, 2015), skill level, and sport type (e.g., Nicholls et al., 2007) differences in coping among athletes. Researchers such as such as Gaudreau (i.e., Gaudreau et al., 2002; Gaudreau and Blondin, 2004b) reported temporal fluctuations in the coping strategies deployed. Although researchers have not compared coping in actual competitions versus laboratory experiments, it is plausible that this could be a moderator variable too, given that athletes are likely to perceive simulated competitions as being less important and stressful (Doron and Gaudreau, 2014). As such, all of these variables may moderate the coping and performance relationship.

### Objectives of current study

As previously mentioned, evaluating and synthesizing the relationship between coping and sporting performance is difficult. This is due to scholars classifying coping differently, reporting coping at the strategy or dimension level, or using different performance measures across diverse sports. In order to accurately compare studies, our first aim was to develop a new coping classification that would make this possible. The second aim was to identify the extent to which each of the coping strategies correlated either positively or negatively with sports performance. Finally, the third aim was to identify whether the effect size between coping and sports performance varied at different levels of a range of evidence-based moderator variables (Ntoumanis et al., 2014).

## Methods

### Information sources and search strategy

We utilized three distinct search strategies to identify appropriate studies. Using the different combinations of keywords (i.e., “coping,” “coping strategies,” “sports performance,” “objective performance,” “subjective performance,” “stress management,” “goal attainment,” and “performance goal discrepancy”) in conjunction with “psychology,” “psychological,” “demographic,” “factors,” “predictors,” “sport,” and “athletes” we searched SportDISCUS, PsycINFO, Google Scholar, PubMed, and Medline for papers. There were no date limits placed on these literature searches. However, only English language based articles were selected for inclusion. Secondly, we manually searched the following journal articles: *International Journal of Sport and Exercise Psychology* (2003–2015), *International Journal of Sport Psychology* (1994–2015), *Journal of Applied Sport Psychology* (1989–2015), *Journal of Clinical Sport Psychology* (2007–2015), *Journal of Sport Behavior* (1990–2015), *Journal of Sport & Exercise Psychology* (1979–2015), *Journal of Sports Sciences* (1983–2015), *Psychology of Sport and Exercise* (2000–2015), *Research Quarterly for Exercise and Sport* (2001–2015), *Sport, Exercise, and Performance Psychology* (2011–2015), and *The Sport Psychologist* (1987–2015). Following the manual search, reference lists of the included papers were searched, which is known as pearl growing (Hartley, 1990).

### Eligibility criteria

**Figure 1:**
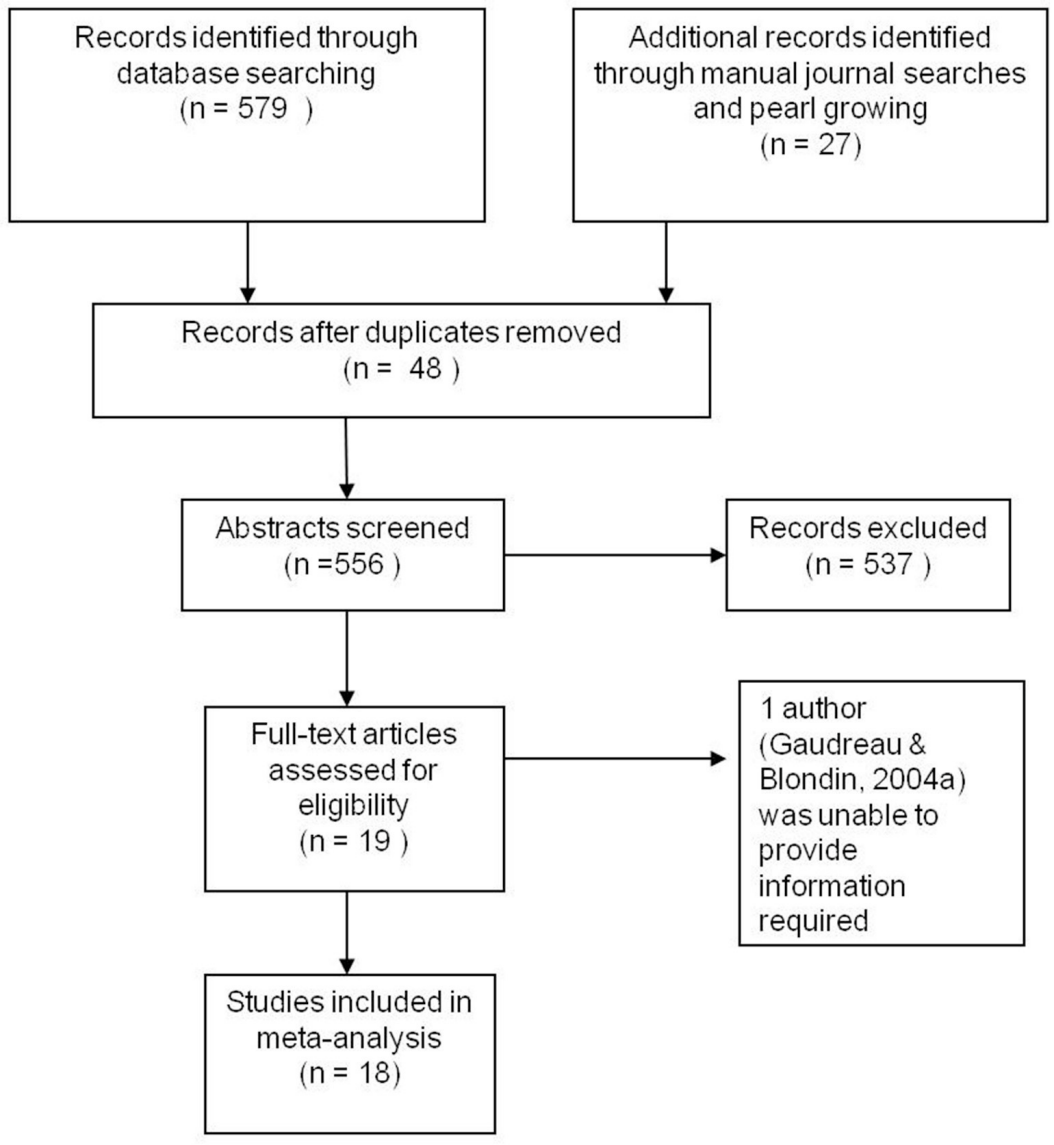
**PRISMA flow diagram**.

Studies published in English language, peer reviewed journals, which assessed the relationship between coping and at least one measure of performance (e.g., objective performance, performance satisfaction, goal attainment, or performance goal discrepancy) were included. In total, 604 records were retrieved in searches, of which 48 were duplicates, so 556 were screened. Based upon the aforementioned criteria, 537 studies were excluded. Accordingly, 19 studies were identified as fulfilling the study inclusion criteria. There were four instances in which the required information for the meta-analysis was not included in the publication and the authors were contacted directly for further information. One study was excluded because the corresponding author was unable to provide the information we required (Gaudreau and Blondin, 2004b). A PRISMA flow diagram illustrating the sequence of dataset selection is depicted in Figure 1. The final pool included 18 independent studies (Haney and Long, 1995; Smith and Christensen, 1995; Amiot et al., 2004; Gaudreau and Blondin, 2004b; Hatzigeorgiadis, 2006; Gaudreau and Antl, 2008; Van Yperen, 2009; Gaudreau et al., 2010; Levy et al., 2011; Nicolas et al., 2011; Nicholls et al., 2012a,b; Schellenberg et al., 2013; Doron and Gaudreau, 2014; Evans et al., 2014; Laborde et al., 2014a,b). A variety of questionnaires, which classified coping in many different ways, were reported in the primary investigations.

### Data collection process/summary measures

Due to the aforementioned limitations of the problem- and emotion-focused and approach and avoidance coping dimensions (e.g., Ayers et al., 1996; Walker et al., 1997; Connor-Smith et al., 2000; Compas et al., 2001) and the difficulty in assigning coping strategies from these dimensions into Gaudreau and Blondin's (2004a) task-, distraction-, or disengagement-oriented coping classifications, we decided that it would be appropriate to devise a new classification. We developed a classification so that coping strategies from all three classifications of coping so widely used in the sport literature (e.g., Lazarus and Folkman, 1984; Roth and Cohen, 1986; Gaudreau and Blondin, 2004a), could be accurately assigned to a specific category. This would permit the data to be analyzed within this meta-analysis and thus shed light on the coping and performance relationship.

The evidence suggests that a two-factor classification of coping is too broad and results in many different coping strategies being classified within the same dimension (Coyne and Gottlieb, 1996; Compas et al., 2001). Further, two-factor classifications of coping do not provide an adequate fit (Ayers et al., 1996), whereas a three-factor approach to coping appears a more valid way of classifying coping (Walker et al., 1997; Connor-Smith et al., 2000). As such, we decided that a three-factor approach would be the most accurate way of classifying coping.

We then examined three-factor classifications of coping from the developmental (e.g., Connor-Smith et al., 2000) and the health psychology literature (e.g., Walker et al., 1997), as these demonstrated excellent model fits. Due to the robust methodological procedures employed by Connor-Smith and colleagues, which involved testing their three-factor sample with two independent samples of 400 and 300 participants, respectively, and the positive results, we decided to base our three-factor classification on their conceptual model. Connor-Smith and colleagues proposed the following three-factor classification: (1) Primary control engagement coping (e.g., solving problems, managing thoughts, and communicate feelings to others), (2) Secondary control engagement coping (e.g., acceptance, avoidance, and positive self-talk) and (3) Disengagement coping (e.g., engaging in different behaviors and thinking about other things).

As the classification of coping proposed by Connor-Smith et al. (2000) was devised and tested for the developmental literature, we adapted the classification so that it would suit the sport literature better. We proposed mastery coping, which was similar to primary control engagement coping, internal regulation that was similar to secondary control engagement coping, and goal withdrawal. Goal withdrawal was similar to disengagement coping. Mastery coping included strategies that involved athletes attempting to take control of a stressful situation and thus eliminate the stressor. Strategies coded within this category included task-oriented and problem-focused coping, engagement, approach, goal setting, mental preparation, concentration, confidence, achievement motivation, coachability, and coping with adversity. Internal regulation involved athletes attempting to manage internal responses to stress and included emotion-focused, acceptance, distraction-oriented, and avoidance coping. Finally, goal withdrawal coping was classified as athletes ceasing in their efforts to achieve a goal. Disengagement-oriented coping, behavioral disengagement, mental disengagement, and venting emotion were categorized as goal withdrawal strategies.

Studies were coded by one member of the research team and were independently coded by the other authors for cross checking. We coded for: (a) gender (male, female, mixed); (b) type of coping strategies measured (mastery, internal regulation, goal withdrawal); (c) type of coping measurement instrument used (e.g., Coping Inventory for Competitive Sport; Gaudreau and Blondin, 2004a), (d) outcome measure (performance, goal attainment, performance-goal discrepancy); (e) type of outcome measure (objective, subjective); (f) time of coping measure (before, during, or after performance); (g) time of outcome measure (before, during, or after performance); (h) type of sport (individual, team, mixed); and (i) experimental conditions (actual competition/performance assessment, laboratory based competition/performance assessment). These variables were considered as potential moderators of observed effects.

### Synthesis of results

Each correlation coefficient of coping and sporting performance, including where the primary studies provided multiple correlations between coping measures and performance, was rounded to two decimal places before being subjected to further calculations. For the main analysis, in which the overall strength of the relationship between coping and performance was assessed, the correlations were coded so that a positive relationship indicated an association between coping and performance in the predicted direction (i.e., mastery coping was associated with better performance). For the purposes of moderator analysis, goal withdrawal correlations were reverted back to their original direction (i.e., if studies found goal withdrawal coping and performance to be negatively related, then this was how it was represented in subgroup analysis) to provide a literal representation of this relationship.

All meta-analyses were conducted in R 3.2.3 (R Development Core Team, 2015) using the package “metafor” (Viechtbauer, 2010) using a random effects model and restricted maximum likelihood (REML) estimation. Separate meta-analyses were conducted for each coping classification (mastery, internal regulation, and goal withdrawal). In each analysis, mean effect sizes (*r*_+_), standard deviations, heterogeneity estimates (*Q* statistic), and percentage of variation accounted for by statistical artifacts (*I*^2^) were computed. Significant *Q*, a *Q* that was larger than the degrees of freedom, and *I*^2^ >50% were taken as indicators of heterogeneity (Higgins et al., 2003). Publication bias was assessed for each model through examination of funnel plots and use of the trim and fill function (Duval and Tweedie, 2000). This function simulates hypothetical studies to account for publication bias and recalculates all estimates.

Sport type (team, individual, mixed), performance measure (objective, subjective), and age were included in a moderator analysis for each coping classification. A moderator variable was considered significant when the 95% confidence intervals (95% CI) around the estimates of effect sizes (*r*_+_) for the different levels of a moderator did not include zero.

To examine how much of the heterogeneity was accounted for by the covariate(s) included in each analysis, the adjusted *R*^2^ was used. Adjusted *R*^2^ is calculated by comparing the baseline value of the heterogeneity variance (τ^2^_*total*_) obtained from the original meta-analysis with the heterogeneity variance from the subgroup or meta-regression analysis (τ^2^_*within*_), using the following formula 1- (τ^2^_*within*_/τ^2^_*total*_; Borenstein et al., 2005).

### Assessment of quality/risk of bias

To account for study biases, the quality of included studies was assessed. A version of Higgins et al.'s (2003) tool was used. This has been adapted and applied to the quality assessment of experimental, cross-sectional, and longitudinal studies (Ntoumanis et al., 2014). Studies were rated within each criterion as low or high risk of bias. A study was rated overall as having a low risk of bias if we scored it low risk on all relevant criteria. We deemed studies with one or more ratings of high risk as having a potential risk of bias. To determine the relevance of the quality assessment criteria, a selection (19%) of studies were coded and reviewed by separate members of the research team. Following discussion between the researchers, it was apparent that one of the criterions was inappropriate for the quality assessment of the studies included in this review. The criteria, participants were randomly selected, was less applicable to the context of this meta-analysis. Many of the authors recruited participants involved in specific sports, or at different levels, so random selection was less relevant to the research design. Therefore, this criterion was not included in our quality assessment. For all other criteria, queries were resolved and ratings were agreed (see Table 1). Level of bias was included in the planned moderator analysis.

**Table 1:** **Risk of bias**.

	**1**	**2**	**3**	**4**	**5**	**6**	**7**	**8**	**9**	**10**	**11**	**12**	**13**	**14**	**15**
Amiot et al., 2004	−	+	+	+	+	+	+								+
Doron and Gaudreau, 2014	+	+	+		+	+	+		+						+
Evans et al., 2014	+	+	+		+	+	+		+						+
Gaudreau and Antl, 2008	−	+	+		+	+	+	−	−						+
Gaudreau et al., 2002	−	+	+		+	+	+		−					+	+
Gaudreau and Blondin, 2004b	−	+	+	+	+	+	+								+
Gaudreau et al., 2010	−	−	+		+	+	+								+
Haney and Long, 1995	−	+	+		+	+	+		+						+
Hatzigeorgiadis, 2006	−	+	+		+	+	+		+	+			+	+	+
Laborde et al., 2014a	−	+	+		+	+	+								+
Laborde et al., 2014b	−	+	+	+	+	+	+	−	+						+
Levy et al., 2011	−	+	+		+	+	+		+						+
Nicholls et al., 2012b	−	+	+		+	+	+								+
Nicholls et al., 2012a	−	+	+		+	+	+								+
Nicolas et al., 2011	−	+	+		+	+	+								+
Schellenberg et al., 2013	−	+	+	+	+	+	+	+	−						
Smith and Christensen, 1995	−	+	+		+	+	+								+
Van Yperen, 2009	−	+	+		+	+	+								+

*The risks of bias of studies included were assessed using the criteria below. Studies were assessed as having (a) no or low risk of bias, or (b) potential risk of bias. Criterion for all studies involved**:** Sampling (1. Participants are randomly selected, 2. Sample sizes are adequate, 3. Participants are representative of various demographic groups, 4. If some participants were excluded from the analyses, the exclusion is justified, 5. When group comparisons were made, participants were matched on other meaningful demographics, and 15. Other risks of bias), and measures (i.e., 6. Validated measures are used, or the authors have provided sufficient supportive information of the psychometric properties of the measures they devised and 7. Measures used were clearly defined and were appropriate). The criterion for studies that adopted a longitudinal or prospective design included: 8. Authors examined whether dropout is random or not and 9. Missing data were treated appropriately. Finally, the following criterion was used for experimental designs: 10. Allocation sequence generated to produce comparable groups. 11. Allocation was concealed, 12. Whether blinding was done and the effectiveness of it, 13. Outcome data for all outcomes were reported. Incomplete outcomes due to attrition and exclusions were addressed, and 14. No selective outcome reporting*.

## Results

### Study characteristics

Eighteen peer reviewed English Language published outputs were included in this meta-analysis. The 18 studies provided fifty-nine separate correlational tests between coping measures and sports performance outcomes (see Table 2). The majority of studies recruited late adolescent or young adult sporting performers with mean participant ages of <22 years. There were two exceptions (Gaudreau et al., 2010; Evans et al., 2014), with these authors recruiting older males (mean 50 years) and mixed gender athletes (mean aged 38 years), respectively. Study sample sizes ranged from 16 to 557 participants [median (IQR) = 135 (301.5)], with 13 published studies each recruiting over 100 participants. Most studies included both men and women. Females represented 57% of the pooled participant cohort. Two studies recruited young females only (Haney and Long, 1995; Doron and Gaudreau, 2014). Researchers recruited participants associated with individual (*N* = 7), team (*N* = 6) and a mixture of individual and team sports (*N* = 5).

**Table 2:** **Summary of studies**.

**Study**	**Sample size (type)**	**Gender**	**Age (m)**	**Type of sport**	**Mastery**	**Internal regu-lation**	**Goal with-drawal**	**Time of coping measure**	**Outcome measure**	**Type of outcome measure**	**Time of outcome measure**	**Conditions**	**Bias**
**COPING MEASURE**													
Amiot et al., 2004	129	Mix	18.0	Mixed	✓		✓	Post	GA	Subjective	Post	Real	Low
Doron and Gaudreau, 2014	16	Female	22.2	Individual	✓			During	P	Objective	During	Lab	Low
Evans et al., 2014	26	Mix	35.8	Individual	✓	✓		Post	GA	Subjective	Post	Lab	Low
Gaudreau and Antl, 2008	186	Mix	18.3	Team	✓	✓	✓	Post	GA	Subjective	Post	Real	Risk
Gaudreau et al., 2002	62	Male	16.4	Individual	✓		✓	Pre; during; post	PGD	Mixed	Post	Real	Low
Gaudreau and Blondin, 2004b	135	Male	27.0	Individual	✓	✓		Post	GA; PGD	Subjective; mixed	Post	Real	Low
Gaudreau et al., 2010	261/316	Male	50.0	Individual	✓	✓	✓	Post	GA; PGD	Objective; subjective	Post	Real	Low
Haney and Long, 1995	178	Female	18.7	Team	✓		✓	Post	P	Objective	Post	Lab	Low
Hatzigeorgiadis, 2006	24	Male	20.3	Individual	✓	✓		Post	P; GA	Objective; subjective	Post; during	Lab	Risk
Laborde et al., 2014a; Study 2	296	Mix	22.6	Mixed	✓	✓	✓	Post	PS	Subjective	Post	Real	Low
Laborde et al., 2014b; Study 2	93	Mix	21.8	Mixed	✓	✓	✓	Post	PS	Subjective	Post	Real	Low
Levy et al., 2011	414	Mix	21.63	Mixed	✓	✓	✓	Post	PS	Subjective	Post	Real	Low
Nicholls et al., 2012b	636	Mix	21.1	Mixed	✓	✓	✓	Post	PS	Subjective	Post	Real	Low
Nicholls et al., 2012a	557	Mix	22.3	Mixed	✓	✓	✓	Post	PS	Subjective	Post	Real	Low
Nicolas et al., 2011	88	Mix	18.5	Individual	✓		✓	Post	GA	Subjective	Post	Real	Low
Schellenberg et al., 2013	421	Mix	19.7	Team	✓		✓	Post	GA; GA change	Subjective	Post	Real	Risk
Smith and Christensen, 1995	104	Male	NS	Team	✓			Pre	P	Objective	Post	Real	Low
Van Yperen, 2009	65	Male	16.6	Team	✓			Post	P	Subjective	NS	Real	Low

Mastery coping was assessed by all studies, internal regulation was assessed by 10 studies, and goal withdrawal was assessed by 12 studies. Five studies measured all three coping strategies. Coping was measured prior to performance (*N* = 14), during performance (*N* = 2), and after performance (*N* = 4). Outcome measures of performance included goal attainment (*N* = 8), goal attainment change (*N* = 1), actual performance (*N* = 5), performance goal discrepancy (*N* = 3), and performance satisfaction (*N* = 5). Subjective performance was measured in 14 studies and objective performance was measured in five studies. A combination of subjective and objective performance was measured in three studies (i.e., those studies in which performance goal discrepancy was assessed). The majority of studies (*N* = 16) assessed performance at the end of the task. Two studies measured performance during performance itself, one study measured performance (goal attainment) prior to performance and in one study the timing of performance measure was not stated. Training or competition conditions were used in 14 studies and controlled laboratory conditions were used in four studies.

### Overall relationship between coping and performance

**Figure 2:**
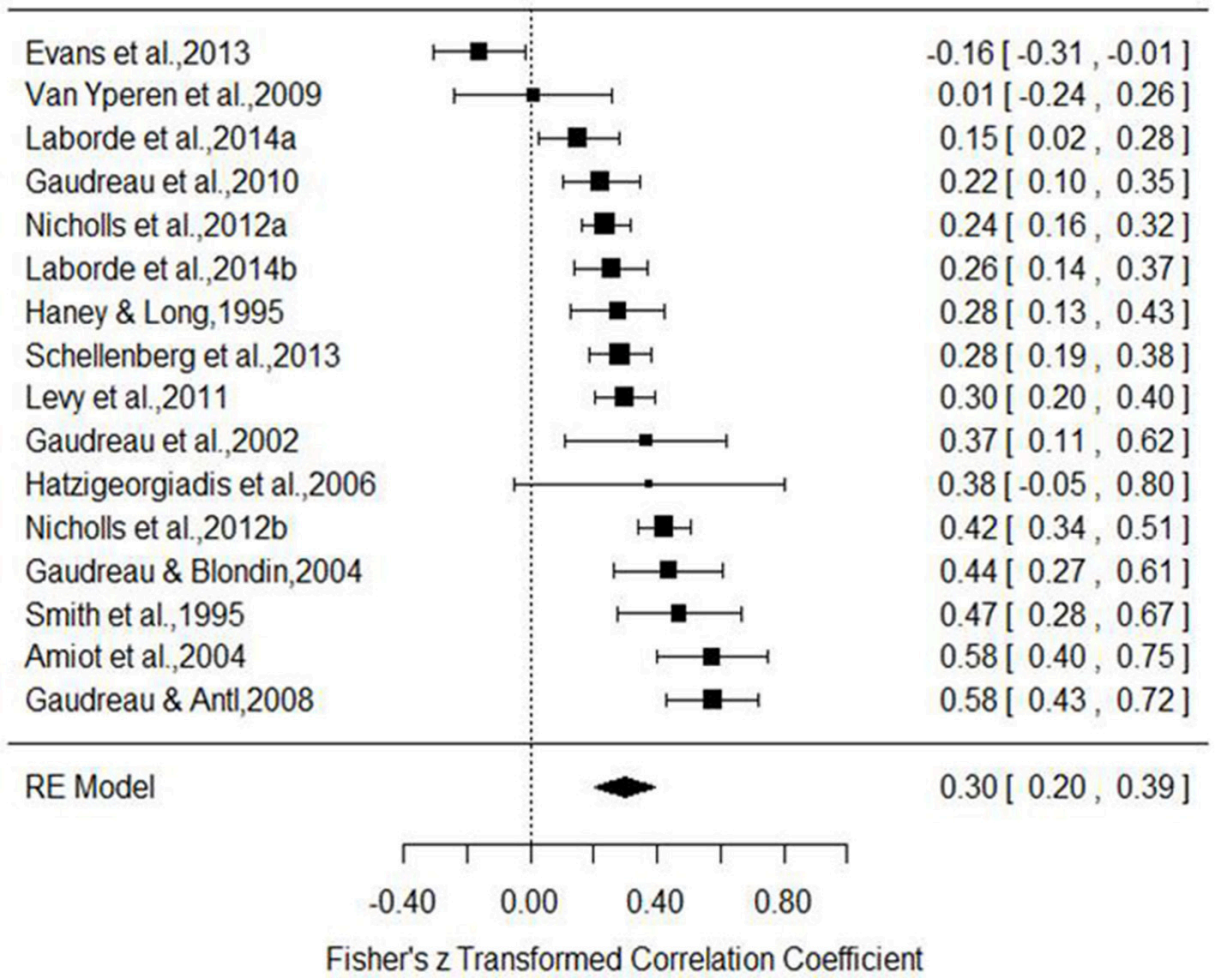
**Mastery coping forest plot**. (Haney and Long, 1995; Smith and Christensen, 1995; Gaudreau et al., 2002, 2010; Amiot et al., 2004; Gaudreau and Blondin, 2004b; Hatzigeorgiadis, 2006; Gaudreau and Antl, 2008; Van Yperen, 2009; Levy et al., 2011; Nicholls et al., 2012a,b; Schellenberg et al., 2013; Evans et al., 2014; Laborde et al., 2014a,b).

**Figure 3:**
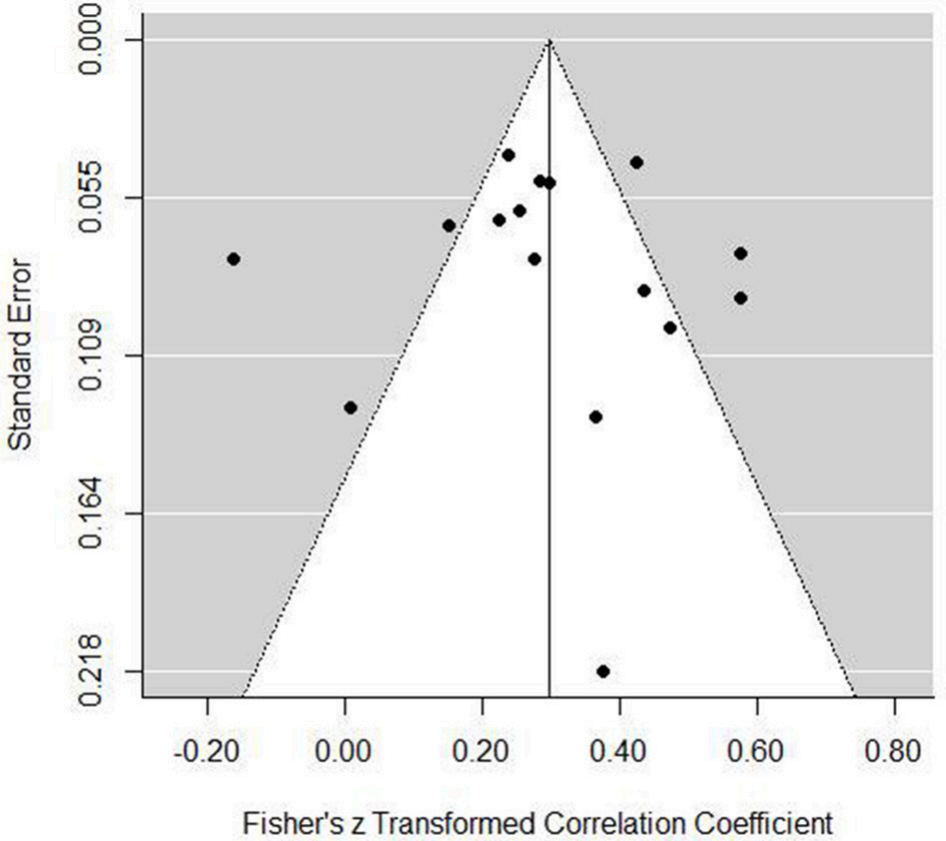
**Mastery coping funnel plot**.

**Figure 4:**
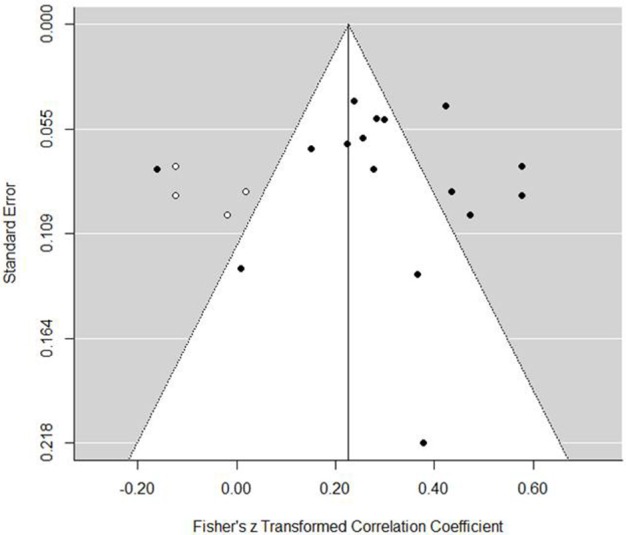
**Mastery coping funnel plot with trim and fill function**.

Grouped coping strategies were examined in three separate models and each tested for moderators. The mastery model indicated a positive association with performance (est. = 0.30, *SE* = 0.05, *z* = 6.20, *p* <0.001, 95% CI = 0.20, 0.39). The forest plot (Figure 2) indicated that one study demonstrated a negative association, one showed no association, and all others were significantly positive. Tests for heterogeneity indicated that a large proportion of variance was not associated with sampling error [*Q*_(15)_ = 90.35, *p* <0.001, *I*^2^ = 87.30%, τ^2^ = 0.030]. To examine potential publication bias, we inspected a funnel plot, which presented a largely well-distributed scatter, but with some deviation from the center (Figure 3). To determine if this impacted on the interpretation of the main finding, we used the trim and fill feature in metafor, which added a further four studies to the left hand side of the funnel (Figure 4). This reduced the overall effect to 0.23 (*SE* = 0.05, 95% CI = 0.13, 0.33) but still demonstrated that, even accounting for publication bias, there is a positive association between mastery coping and performance. We next tested several moderators to determine any potential impact on the observed association. Specifically, we tested sport type (team, individual, or mixed), performance measure (objective or subjective), and age (Table 3). The Knapp and Harting adjustment (KNHA) was included to provide a conservative approach to moderation estimation, as number of studies is relatively low. Overall, there was no strong evidence for the effect of these as significant moderators of the relationship between mastery-based coping and sports performance.

**Figure 5:**
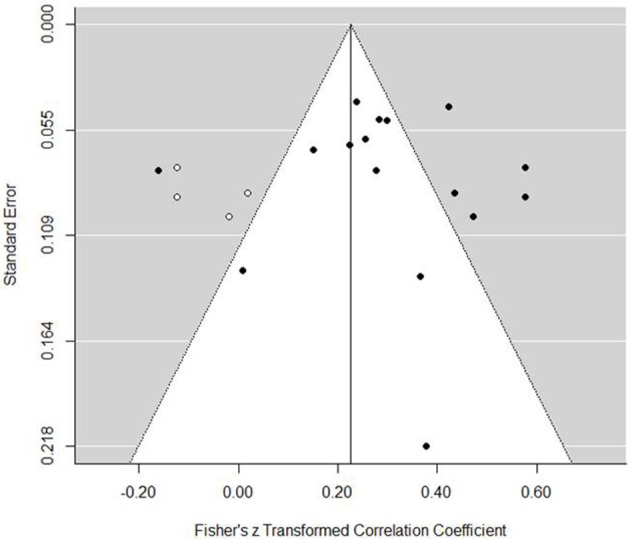
**Internal regulation coping forest plot**. (Gaudreau and Blondin, 2004b; Hatzigeorgiadis, 2006; Gaudreau and Antl, 2008; Gaudreau et al., 2010; Levy et al., 2011; Nicholls et al., 2012a,b; Evans et al., 2014; Laborde et al., 2014a,b).

**Figure 6:**
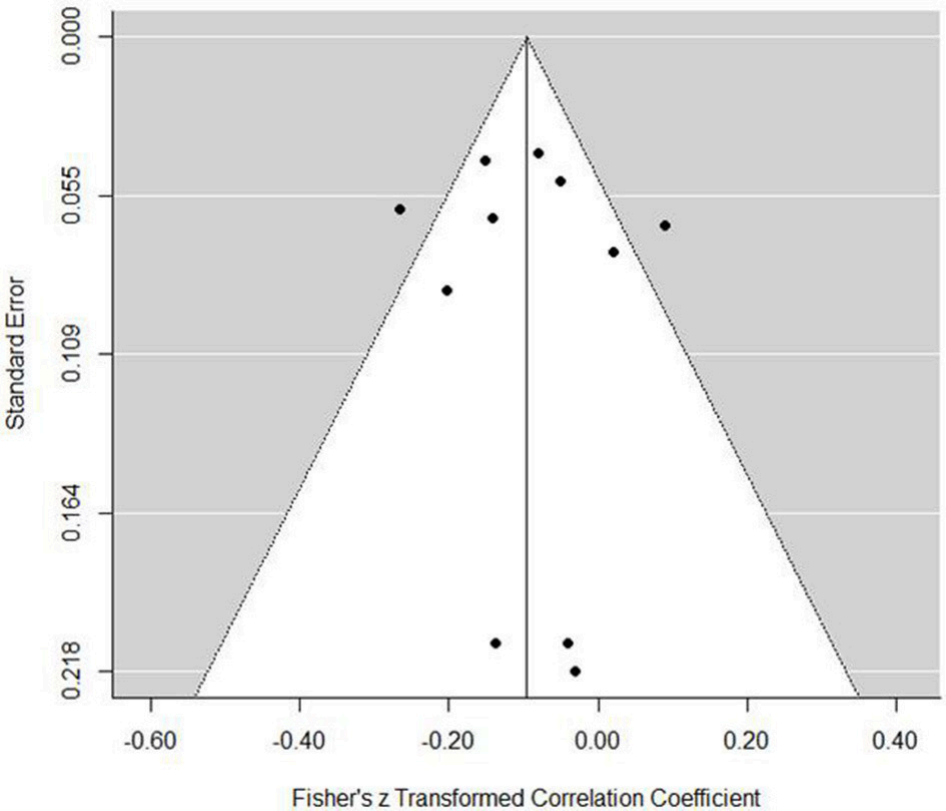
**Internal regulation coping funnel plot**.

The internal regulation model presented a small but statistically significant negative association with performance (est. = −0.10, *SE* = 0.04, *z* = −2.66, *p* = 0.008, 95% CI = −0.17, −0.03). The forest plot (Figure 5) demonstrates a small negative association although only four of the 11 studies included present confidence intervals with an absence of zero. In a practical sense, the association is negligible. Further evidence of the caution with this result was found in tests for heterogeneity, which indicated that a reasonable proportion of variance can be explained through chance [*Q*_(10)_ = 24.03, *p* = 0.008, *I*^2^ = 64.23%, τ^2^ = 0.008]. Publication bias was not an issue for internal regulation and performance associations (Figure 6). Indeed, the trim and fill function only added two evaluations, making little difference to the prediction (est. = −0.13, *SE* = 0.04, 95% CI = −0.21, −0.06). Moderators indicated no significant effect (Table 3).

**Table 3:** **Moderator analyses**.

**Classification**	**Moderators**	***I*^2^**	***T*^2^**	***k***	**Est. (95% CI)**	***QE***	***H*^2^**
Mastery		87.30%	0.030	16	0.30 (0.20, 0.39)	90.35^***^	
	**Sport type**						
	Individual			5	0.59 (−0.14, 1.31)		
	Team			5	0.47 (−0.12, 1.06)		
	Mixed			6	0.57 (−0.02, 1.16)		
	**Performance measure**						
	Objective			5	0.18 (−0.89, 1.27)		
	Subjective			11	0.04 (−0.79, 0.86)		
	Age			16	−0.01 (−0.05, 0.02)		
		92.60%	0.045	14		72.22^***^	13.51%
Internal Regulation		64.23%	0.008	11	−0.10 (−0.17, −0.03)		
	**Sport type**						
	Individual			5	−0.07 (−0.83, 0.70)		
	Team			1	0.10 (−0.56, 0.75)		
	Mixed			5	−0.01 (−0.68, 0.65)		
	**Performance measure**						
	Objective			2	−0.06 (−0.22, 0.34)		
	Subjective			9	−0.06 (−0.43, 0.32)		
	Age			11	−0.00 (−0.02, 0.02)		
		75.26%	0.011	11		20.12^**^	4.04%
Goal Withdrawal		89.13%	0.030	12			
	**Sport type**						
	Individual			3	−0.38 (−0.99, 0.23)		
	Team			3	−0.57 (−1.18, 0.05)		
	Mixed			6	−0.30 (−0.96, 0.36)		
	**Performance measure**						
	Objective			3	0.49 (−0.36, 1.33)		
	Subjective			9	0.26 (−0.51, 1.02)		
	Age			12	−0.01 (−0.03, 0.02)		
		93.15%	0.040	12		56.64^***^	14.60%

*k, number of evaluations; H^2^, Heterogeneity accounted for by moderator*.

** *p <0.01*,

*** *p <0.001*.

**Figure 7:**
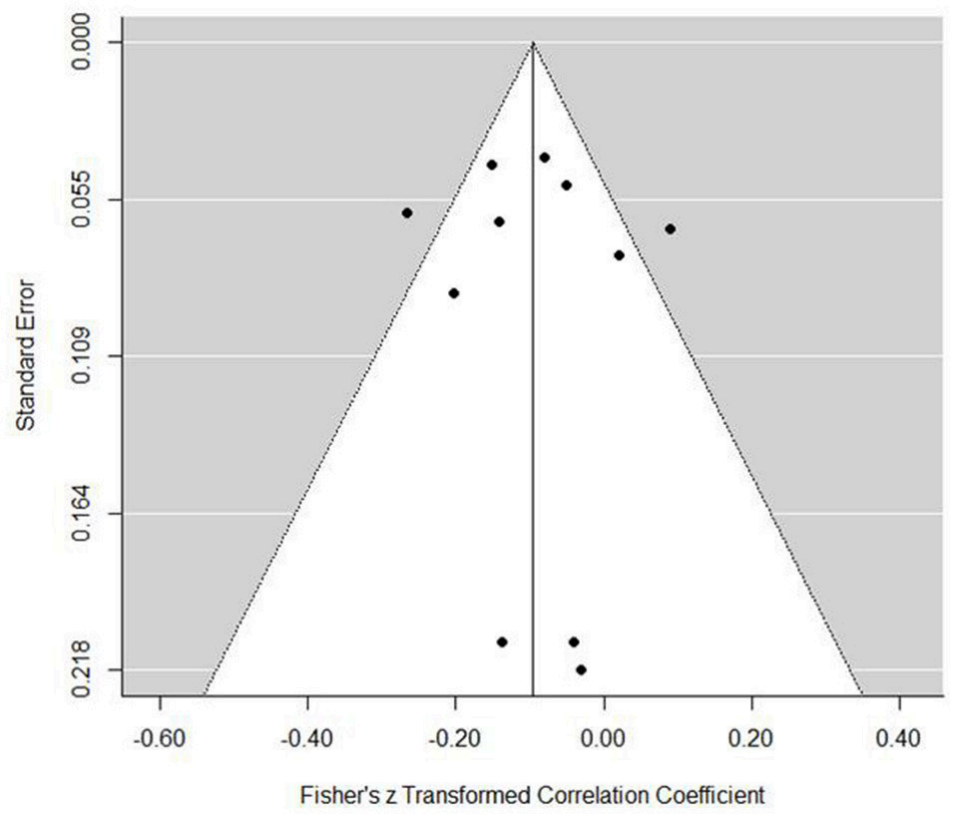
**Goal withdrawal coping forest plot**. (Haney and Long, 1995; Gaudreau et al., 2002; Amiot et al., 2004; Gaudreau and Antl, 2008; Gaudreau et al., 2010; Levy et al., 2011; Nicolas et al., 2011; Nicholls et al., 2012a,b; Schellenberg et al., 2013; Laborde et al., 2014a,b).

**Figure 8:**
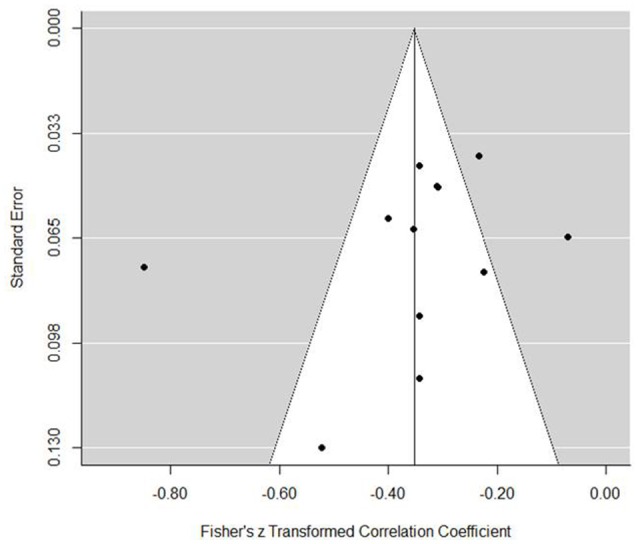
**Goal withdrawal coping funnel plot**.

In terms of goal withdrawal, the model indicated a significant negative effect (est. = −0.35, *SE* = 0.06, *z* = −6.55, *p* <0.001, 95% CI = −0.46, −0.25). The forest plot (Figure 7) provides a clear trend, in that all studies except one included demonstrated a significant negative relationship between goal withdrawal and performance. Heterogeneity tests supported that variance was not overly associated with sampling error [*Q*_(11)_ = 77.32, *p* <0.001, *I*^2^ = 89.13%, τ^2^ = 0.030]. Tests for publication bias indicated that there was little concern (Figure 8). Indeed, trim and fill analysis did not add any additional studies. Tests for moderators indicated no significant effect (Table 3).

## Discussion

This is the first meta-analysis to assess the relationship between coping and sporting performance. Our overreaching objective was to synthesize data from independent samples in order to examine the relationship between different coping strategies and sports performance. We also aimed to evaluate factors that might be associated with this relationship, such as gender, sport type, participant skill level, time of coping assessment, and actual training/sporting versus laboratory-based testing conditions.

The first aim of the study was to create a generalized system to categorize the different coping strategies. Our new classification of coping was based on Connor-Smith et al. (2000), but adapted for the sport psychology literature. This resulted in us creating mastery, internal regulation, and goal withdrawal strategies. We purposely selected a three-factor model as it demonstrates a better model fit than a two-factor classification of coping (e.g., Ayers et al., 1996). Our classification of coping allowed us to categorize studies that used either the problem- and emotion-focused (Lazarus and Folkman, 1984), approach and avoidance (Roth and Cohen, 1986), or Gaudreau and Blondin's (2004b) three-factor classification of coping. In terms of future research, the three-factor model of coping that we proposed or that by Gaudreau and Blondin (2004a) appears a more accurate way of classifying coping in comparison to either the problem- and emotion-focused or approach and avoidance classifications of coping. Indeed, scholars could develop a coping questionnaire that classifies coping in the three-factor we used in this meta-analysis, and thus includes master, goal withdrawal, and internal regulation strategies. This questionnaire could assess coping at the strategy level too, so that researchers can assess coping at the dimensional or strategy level, depending on their research question.

The overall correlation between coping and sporting performance was shown to be significant and heterogeneous. Mastery coping was positively associated with sports performance, whereas goal withdrawal was negatively associated with performance. Internal regulation was not shown to be significantly associated with sports performance in the 10 studies that evaluated this construct. The positive correlation between mastery coping and performance is aligned to the relationship between coping and coping effectiveness. Researchers found that mastery type strategies tended to be the most effective at reducing stress levels, whereas goal withdrawal strategies were negatively associated with coping effectiveness (Nicholls et al., 2010). Given the cross-sectional nature of much of the research, it remains unclear whether employing mastery coping reduces stress, which in turn facilitates performance, or whether mastery coping independently facilitates performance, which in turn causes athletes to view their coping as being more effective. The interplay between coping and performance, along with perceptions of effectiveness requires more attention. Further, it is important that scholars identify the most effective mastery coping strategies, because there were some studies that showed no or a negative association with sports performance. Due to the lack of studies, it was not possible to conduct separate analyses to assess the relationship between individual coping strategies and sport performance.

We found a negative effect for the relationship between goal withdrawal strategies and performance. There is conflicting evidence regarding goal withdrawal strategies across different domains of psychology. In the mental health literature, Wrosch et al. (2011) found that withdrawal strategies may be helpful for enhancing well-being among a sample of caregivers, whereas Nicholls et al. (2016c) reported that such strategies were negatively associated with well-being among an athletic sample. These contrasting findings may be due to the nature of the stress among the participants of the two studies. The caregivers in the Wrosch sample were dealing with chronic stressors, whereas the athletes in the Nicholls sample were contending with acute stressors. Further research is required to unpick the factors that may impact on the relationship between withdrawal and well-being. Nevertheless, the present study indicates that this strategy is not helpful to sports performance and it may also be detrimental to the well-being of athletes.

The observed effect sizes relating coping to sporting achievement represent statistical, but not causal effects. Some scholars may argue that the limited number of studies and experimental research, in particular, are limitations of this meta-analysis. We, however, hold a different view. Firstly, the Cochrane database of systematic reviews includes over 3000 reviews. Most of these reviews are randomized controlled trials. The median number of trials included in a review, however, was six (Borenstein et al., 2009). Although the number of studies in this review was low in comparison to other meta-analyses (e.g., Ntoumanis et al., 2014), this number was notably higher than six, and each study performed a range of correlations, providing our meta-analysis with 59 tests to assess. Secondly, even though experimental research designs allow researchers to infer causality, it is unlikely that stress levels experienced within laboratory settings would be similar to those generated in actual competitions (Doron and Gaudreau, 2014). This is because actual competitions have real consequences for athletes, such as whether they will receive a new contract, attain selection for a team, win a competition, or even maintain a sponsorship deal. There is undoubtedly a trade-off between inferring causality and assessing how athletes cope when managing ecologically valid stressors. Similar to Ntoumanis et al.'s (2014) meta-analysis, we were unable to test all of the moderator variables, where appropriate, for each study. This is due to data not being consistently reported within the primary studies for the relationship between coping and performance across the different sub-groups. Further, based up publication bias, the findings regarding internal regulation should be interpreted with caution.

On the basis of our findings, participants could be taught and encouraged to use mastery coping strategies across all phases of competitive sporting events. Using mastery strategies may help athletes increase their performance levels, regardless of how performance is assessed. Athletes could also be instructed not to use goal withdrawal strategies during competitive events. Finally, athletes could be discouraged from using internal regulation to maximize performance. Future research could test our recommendations. Reeves et al. (2011) examined the effects of a coping intervention on subjective performance, but we could not include this in our meta-analysis because it was qualitatively based. As such, researchers could quantitatively examine the effects of coping interventions on both objective and subjective performance in order to address causality. Previous research, albeit a case study with an elite golfer (Nicholls, 2007) provided tentative support for the notion that athletes can be taught to use particular strategies and refrain from using other strategies. It should also be noted, however, that changing the way an athlete copes will not be instant fix and may require time and effort on the part of both the sport psychologist and athlete (Nicholls, 2007). Nevertheless, making such changes could be beneficial to performance.

In conclusion, we found a significant relationship between coping strategies and sports performance. In particular, mastery based coping strategies were positively associated with performance, whereas internal regulation and goal withdrawal was negatively associated with performance. Practitioners could encourage athletes to use mastery based coping strategies, although future research is required, however, to identify the mastery strategies that will have the most beneficial impact upon sporting performance, as there were anomalies between some of the studies. Due to number of studies within this meta-analysis, we were unable to examine the relationship between individual mastery coping strategies and performance. Identifying the relationship between individual mastery coping strategies and performance will make a worthy contribution to the current sport psychology literature. Additionally, it would also be interesting to examine the effectiveness of different coping strategies on performance among athletes of different ages and gender, because if there are differences coping interventions would need to be devised specifically for the population in mind.

## Author contributions

AN, NT, SC, and JP contributed to the writing of the manuscript. NT and JP performed the statistical analyses.

### Conflict of interest statement

The authors declare that the research was conducted in the absence of any commercial or financial relationships that could be construed as a potential conflict of interest.
